# The signal peptide-like segment of hpaXm is required for its association to the cell wall in transgenic tobacco plants

**DOI:** 10.1371/journal.pone.0170931

**Published:** 2017-01-31

**Authors:** Le Li, Weiguo Miao, Wenbo Liu, Shujian Zhang

**Affiliations:** 1 College of Environment and Plant Protection, Hainan University, Haikou, Hainan Province, China; 2 Hainan Key Laboratory for Sustainable Utilization of Tropical Bioresource, Haikou, Hainan Province, China; 3 U.S. Horticultural Research Laboratory, USDA-ARS, Fort Pierce, Florida, United States of America; Instituto de Biologia Molecular y Celular de Plantas, SPAIN

## Abstract

Harpins, encoded by *hrp* (hypersensitive response and pathogenicity) genes of Gram-negative plant pathogens, are elicitors of hypersensitive response (HR). HpaXm is a novel harpin-like protein described from cotton leaf blight bacteria, *Xanthomonas citri* subsp. *malvacearum—*a synonym of *X*. *campestris* pv. *malvacearum* (Smith 1901–1978). A putative signal peptide (1-MNSLNTQIGANSSFL-15) of hpaXm was predicted in the nitroxyl-terminal (N-terminal)by SignalP (SignalP 3.0 server). Here, we explored the function of the N-terminal leader peptide like segment of hpaXm using transgenic tobacco (*Nicotiana tabacum* cv. Xanthi nc.). Transgenic tobacco lines expressing the full-length *hpaXm* and the signal peptide-like segment-deleted mutant *hpaXmΔLP* were developed using transformation mediated by *Agrobacterium tumefaciens*. The target genes were confirmed integrated into the tobacco genomes and expressed normally. Using immune colloidal-gold detection technique, hpaXm protein was found to be transferred to the cytoplasm, the cell membrane, and organelles such as chloroplasts, mitochondria, and nucleus, as well as the cell wall. However, the deletion mutant hpaXmΔLP expressed in transgenic tobacco was found unable to cross the membrane to reach the cell wall. Additionally, soluble proteins extracted from plants transformed with *hpaXm* and *hpaXmΔLP* were bio-active. Defensive micro-HR induced by the transgene expression of hpaXm and hpaXmΔLP were observed on transgenic tobacco leaves. Disease resistance bioassays to tobacco mosaic virus (TMV) showed that tobacco plants transformed with *hpaXm* and with *hpaXmΔLP* exhibited enhanced resistance to TMV. In summary, the N-terminal signal peptide-like segment (1–45 bp) in *hpaXm* sequence is not necessary for transgene expression, bioactivity of hpaXm and resistance to TMV in transgenic tobacco, but is required for the protein to be translocated to the cell wall.

## Introduction

Harpins, encoded by hypersensitive response and pathogenicity (*hrp*) genes of Gram-negative plant-pathogenic bacteria, are glycine-rich, heat stable, cysteine-free, protease-sensitive, acidic proteins that share common characteristics [1]. They can induce hypersensitive response (HR) and systemic acquired resistance in non-host plants through exogenous application [2]. It was reported that several harpins expressed in transgenic plants including tobacco, rice, and *Arabidopsis* induced host responses such as enhanced growth and drought tolerance [3–7]. According to their general characteristics, harpins are categorized into four major groups based on their protein similarity and domain structures: HrpN, HrpZ1, HrpW1, and Hpa1 groups [1]. HpaXm is a newly found harpin-like protein, cloned and identified from cotton leaf blight bacteria, *Xanthomonas citri* subsp. *malvacearum—*a synonym of *X*. *campestris* pv. *malvacearum* (Smith 1901–1978). Based on its composition of amino acids and other characteristics, hpaXm was distinct from other harpins and was classified into a new group [1]. Previous works showed that hpaXm was capable of inducing HR in plants and enhancing resistance to tobacco mosaic virus (TMV) in tobacco when infiltrated into the leaf apoplast using purified protein [8].

Over the last few decades, it was found that most harpins travel through the type III secretion system in pathogens. The translocation of some type III secretion effectors into plant cells relies on the effector targeting signal that generally resides in the nitroxyl-terminal (N-terminal) domain [9–10]. In general, studies on the N-terminal function of harpins have been mainly described in bacteria, including several plant pathogenic bacteria, and have focused on the regions responsible for eliciting the HR reaction or controlling systemic resistance. For example, a conserved N-terminal region consisting of 12 highly hydrophilic amino acids (QGISEKQLDQLL) played a critical role in protein aggregation and inclusion body formation in harpins from *Xanthomonas* [11]. Additionally, the dual effect depends on plants’ sensing of the N-terminal region in the Hpa1 sequence [12–13], as the region of 10–42 residues (Hpa1_10–42_) in the Hpa1 sequence is 1.3–7.5-fold more effective than the full length in inducing plant resistance [13–16]. However, in tobacco, Hpa1_10–42_ is near 30-fold less active than Hpa1 in eliciting hypersensitive cell death (HCD) [16–17]. Previous works regarding the function of the N-terminal amino acids of different harpins revealed that the N-terminal leader peptide of several harpins is critical for their efficacy [1,18–19]. But there was few research on the relationship between the N-terminal amino acids of harpin and its translocation in plant tissues.

In particular, signalP (SignalP 3.0 server; http://www.cbs.dtu.dk/services/SignalP/) prediction suggested that a putative signal peptide of hpaXm located in the N-terminal leader peptide (1-MNSLNTQIGANSSFL-15). Furthermore, glutathione S-transferase (GST) trap test indicated that hpaXm was released into the extra-cellular medium, and hpaXmΔLP (the mutation of the N-terminal signal peptide-like segment deletion hpaXm) recombinant protein was unable to be secreted outside cells but still elicited a HR reaction in tobacco leaves [8, 20–22]. The exogenously expressed protein involving a cleavable N-terminal signal peptide, which is cleaved off during translocation across the cytoplasmic membrane via the Sec translocation (Sec) or twin-arginine translocation (Tat) machinery [23–24], is thought to be exported by the Type II secretion system (T2SS) [23]. The presumed signal peptide suggested that hpaXm was probably distinct from other harpins, probably transported with T2SS [1,20–22]. Previous works [8] showed that the leader peptide (1-MNSLNTQIGANSSFL-15) in the N-terminal region of hpaXm was probably the key factor relating to the hpaXm’s pathogenicity capability, association sites with host or non-host plant tissues, intracellular secretion and translocation in bacteria. Therefore, previous studies on the function of the leader peptide in the N-terminal region of hpaXm have mainly targeted bacteria such as *Xanthomonas* pathogens or *E*. *coli*. [20]. Whether or not the N-terminal leader peptide influences the trans-gene expression of hpaXm in transgenic plants remains unclear.

Another consideration is the association sites of hpaXm with plants, especially the transgene expression of hpaXm in plants. It was shown that translocator proteins in animal pathogenic bacteria can form a channel-like structure in the host plasma membrane by oligomerization [1, 25]. For example, HrpZ1 from *Pseudomonas syringae* pv. *phaseolicola* was shown to be associated with liposomes and synthetic bilayer membranes [26]. Moreover, PopA1 from *Ralstonia solanacearum* was shown to be integrated into liposomes and membranes of *Xenopus laevis* oocytes, resulting in the formation of ion-conducting pores [27]. However, it was reported that PopW could target the plant cell wall and bind to calcium pectate [28]. Harpin_Xoo_ expressed in transgenic cotton plants was also found to be associated with the cell wall [21–22]. Although some studies have suggested that several harpins were associated with plant cell membranes, as they could bind to lipids and form pores in the plant, the association sites of harpins in transgenic plants remain controversial. Whether or not the N-terminal regions of harpins influence the association sites, the interaction sites and the translocation of harpins in plants also remains unclear.

In this work, we studied the full-length hpaXm and the signal peptide-like segment-deleted mutant hpaXmΔLP using transgenic tobacco plants. Transgenic tobacco expressing *hpaXm* or *hpaXmΔLP* genes were developed and the bio-activity of the extracted soluble proteins, resistance to TMV of transgenic tobacco plant were determined. Our results suggested that the N-terminal signal peptide-like segment of *hpaXm* is not necessary for the bioactivity of hpaXm and its function in disease resistance to TMV in transgenic tobacco, but is required for the protein to be translocated to the cell wall.

## Materials and methods

### Plants and microbes

Tobacco (*N*. *tabacum*) cv. Xanthi nc and its transgenic lines were grown in a greenhouse for 38 days before use, except specified elsewhere. TMV was maintained in an aqueous solution of 18 mg ml^−1^ at 4°C. *Agrobacterium tumefaciens* strain LBA4404, harboring the binary vector pBI121 [29], was used in construction of the transformation units. The *E*. *coli* strain BL21 (DE3), containing the prokaryotic expression vector BL21/pGEX-hpaXm [20], was used to prepare active control protein hpaXm. Purified protein of hpaXm was lyophilized and maintained at −80°C. Bacteria were multiplied before use as previously described [29–31].

### Generation of transgenic plants

A full-length and the N-terminal leader peptide deleted mutant of *hpaXm* gene were excised from BL21/pGEX-hpaXm and inserted into pBI121 between the CaMV 35S promoter and the β-glucuronidase gene *uidA* by digestion with restriction enzymes *Xba*I and *Xma*I and ligation with T4 DNA ligase [32]. The resulting *pBI-hpaXm* and *pBI-hpaXmΔLP* constructs were sequenced to verify the correct orientation of genes and were transferred into *A*. *tumefaciens* LBA4404 using the standard freeze–thaw protocol [33]. Tobacco was transformed with *pBI-hpaXm*, *pBI-hpaXmΔLP*, or pBI121 by soaking leaf disks with the appropriate *A*. *tumefaciens* suspensions [20]. Tissue culture and plant regeneration were done as previously described [32]. Seed from the T_0_ and T_1_ generations were screened on Murashige and Skoog (MS) agar medium [30] in the presence of kanamycin at 150 μg ml^−1^ [34–35]. After genomic integration, expression, and production of the target genes in transgenic plants were determined, the *hpaXm*- and *hpaXmΔLP*-expressing tobacco plants were compared with untransformed plants in height and weight of the entire plant, weight and size of leaves, and flowering time. Plants containing the vector pBI121 only were similarly selected and designated as Vec lines.

### Nucleic acid manipulation

Standard protocols [32,36] were used. RNA and genomic DNA were isolated from leaves. PCR and DNA blot analyses were conducted for integration of *hpaXm* and *hpaXm△LP* into tobacco genomes. Expression of the trans-gene and defense-related genes were studied by reverse transcription (RT)-PCR or RNA gel blot analyses. The *hpaXm* and *hpaXmΔLP* cDNA used as probes in DNA and RNA blot analyses were produced by PCR from *E*. *coli* strain BL21/pGEX-hpaXm. Sequences of PCR and RT-PCR products were confirmed as described [37]. Various nucleic acids were fractionated by electrophoresis in agarose gels and visualized by staining with ethidium bromide, using DL2000 markers (Takara Biotech Co., Dalian, China) to indicate size. In Southern blot hybridization, 3 μg of genomic DNA was digested with *EcoR*I and *BamH*I prior to electrophoresis and, after that, blotted to nylon membranes. Replicates of blots were hybridized with a digoxigenin-labeled *hpaXm* probe [36]. PCR was conducted for 30 cycles using 3 μg of genomic DNA as a template and specific primers, producing an intact *hpaXm* sequence and an intact *hpaXmΔLP* sequence, respectively. For RT-PCR, reaction conditions were optimized for each gene [37]. RNA (2 μg) was treated with RNase-free DNase and used as template to synthesize the first-strand cDNA. An equal volume of cDNA was amplified for 30 cycles using specific primers (Table 1). The *EF1a* gene was used as a standard and amplified with specific primers, resulting in a 495-bp product [37]. Products were quantified in gels by a scanner attached to an Image System (SX-100; Shanghai Sixing Biol. Tech. Co., Shanghai, China).

**Table 1:** Information of genes and primers for PCR analyses.

Gene	Accession No.	Primer Sequence	Product Size
*hpaXm*	DQ643828	5'-GCTCTAGAATGAATTCTTTGAACACACAGA -3'	420 bp
		5'-TCCCCCCGGGTTACTGCATCGATCCGGTGT-3'	
*hpaXm△LP*	DQ643828	5'-GCTCTAGAATGCAGGTCGACCCGAGCCAGA-3'	360 bp
		5'-TCCCCCCGGGTTACTGCATCGATCCGGTGT-3'	
*35S*	AF485783	5'-gccttttcaatttcagaaagaatgc -3'	800 bp
		5'-ttatatagaggaagggtcttgcgaa -3'	
*EFlα*	AJ223969	5'-AGACCACCAAGTACTACTGCAC-3'	495bp

Note: Restriction site are underlined.

### Protein analysis and micro-HR observation

Soluble proteins were isolated from leaves as described [38] with several modifications. The protease inhibitor phenyl-methylsulfonyl fluoride (PMSF), which protects harpins from destruction by proteases [39], was applied at 0.1 mmol l^−1^ to the protein extraction solution. Supernatants from centrifuging (25,000 × *g* for 25 min at 4°C) leaf homogenates were heated in a boiling water bath for 10 min, cooled to room temperature, and centrifuged similarly. The second supernatants were supplemented with PMSF and maintained overnight at −4°C. After centrifuging, the third supernatants were lyophilized and maintained at −80°C. The protein preparations were tested for bioactivity in aqueous solutions. Purified proteins were resolved by SDS-PAGE. Proteins were also isolated from intercellular spaces of leaves, as described [39] in the presence of PMSF, lyophilized, and maintained as above. Partial aqueous solutions of the protein preparations were heated in a boiling water bath for 10 min, while another part remained untreated; both were tested for bioactivity and resolved by a native PAGE.

Bioactivity of the two types of plant proteins was boiled for 10 min [20] and evaluated, in comparison with hpaXm proteins [40] from *E*. *coli* strain BL21/pGEX-hpaXm, based on the occurrence of HR in tobacco leaves infiltrated with each of protein preparations at 100 μg ml^−1^, a concentration sufficient for the response [37].

Micro-HR was monitored based on observing dead cells after staining leaves with lactophenol trypan blue [37, 41–42]. Briefly, 1.5-cm squares of tobacco leaves were placed in lactophenol trypan blue solution, composed of 10 ml of lactic acid (85% aqueous), 10 ml of water saturated phenol, 10 ml of glycerol (98%), 10 ml of distilled water, and 15 mg of trypan blue. The dye solution was infiltrated into the leaf intercellular spaces with the aid of a vacuum pump and a bell jar. The infiltrated tissues were then heated in a boiling water bath for 5–10 min and incubated at room temperature for 6–8 h. Stained leaf tissues were cleared in chloral hydrate solution (2.5 g ml^−1^) overnight or until tissues were clear. To determine whether both responses spontaneously occurred in transgenic lines, every third leaf on the plants was investigated similarly at 10-d intervals until flowering.

### Disease scoring

TMV was maintained in an aqueous solution (18 mg ml^−1^) at 4°C [29]. Plants were inoculated respectively with TMV at 38 days after transplanting plants to soil. Inoculation was performed for TMV (100μL; 18 μg ml^–1^ of solution) by rubbing leaves using a finger in the presence of abrasive diatomaceous earth on each of six sites of tobacco leaf surfaces [5, 29, 43]. Inoculated plants were grown in a greenhouse maintained at room temperature (20°C–23°C) and investigated for infection 7 d later [3, 29].

For plants inoculated with TMV, the number and size of lesions on leaves were determined. Disease severity (DS) was expressed as number of lesions per leaf for TMV. Correspondingly, resistance was given as percent decrease of DS in transgenic plants, relative to that in untransformed or Vec plants, quantified using the formula 100 × (mean DS in control plants—mean DS in transgenic plants)/mean DS in the control [3].

### Statistical analysis

Assays were conducted three times, each involving 20 plants, except if specified in figure captions. For all quantitative determinations, data were analyzed by Duncan’s multiple range test at *P* ≤ 0.01 [3]. For differences in DS, each transgenic line was compared with Vec plants; T1-101-3 line, T1-103-5 line, and V35 line were also subjected to multiple comparisons.

### Immune colloidal-gold testing

Polyclonal antibody (pAb) against hpaXm were customized from Abmart Company (Shanghai, China), by injecting two New Zealand white rabbits respectively with the purified (≥ 3 mg) hpaXm protein [20] for three times at 2–3-week intervals. Blood containing the antiserum was collected 2 weeks after the final injection.

Colloidal-gold was preserved in our laboratory. Leaf samples of the tested transgenic lines expressing the full-length and the N-terminal leader peptide deleted mutant of *hpaXm* were fixed, washed, dehydrated, penetrated, embedded, and polymerized, with the tested vector-expressing transgenic line as control [20, 44]. Then the leaf samples were cut into about 6-nm-thick slices and adsorbed on copper web under the ultramicrotome of Leica-EM UC7 [18, 44]. Copper webs containing the leaf samples were moist, closed, and incubated with the diluted hpaXm-pAb twice [18, 44]. After being washed and dyed, copper webs were observed under a Hitachi H-7650 electron microscope [18, 44]. All tested samples of each group were repeated at least twice.

## Results

### The exogenous genes were integrated into genomes and expressed normally at the transcriptional level

**Fig 1:**
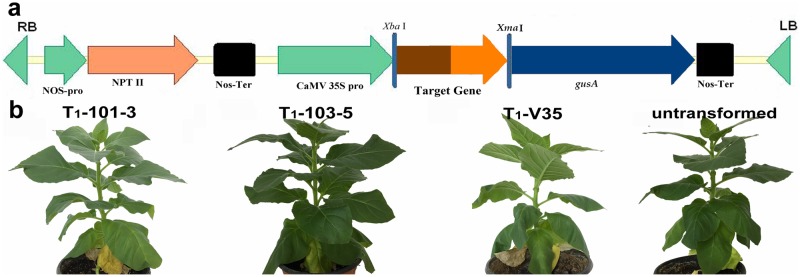
Generation of transgenic tobacco expressing the full-length or the signal peptide-like segment of hpaXm. **a)** The transformation unit *pBI-HpaXm* or *pBI-HpaXmΔLP* constructed in the vector pBI121. Labels on the bottom include the kanamycin-resistant gene *NPT II*, the *35S* promoter, and target gene the full-length *hpaXm* (420 bp) or the N-terminal leader peptide (1-MNSLNTQIGANSSFL-15) deletion mutant *hpaXmΔLP* (357 bp). Those on top are restriction sites *Xba*I and X*ma*I. **b)** Morphological comparison among T_1_ plants of the *hpaXm*-expressing tobacco (T_1_-101-3), the *hpaXmΔLP*-expressing tobacco (T_1_-103-5), the Vec-expressing tobacco (T_1_-V35), and the parent untransformed plants. All the experiments each involving 15 plants gave similar results at least.

The *hpaXm* and *hpaXmΔLP* genes were introduced into tobacco (*Nicotiana tabacum* cv. Xanthi nc.) plants by *Agrobacterium*-mediated transformation. In our previous studies, *Agrobacterium* transformants containing the pBI121::*hpaXm* and pBI121::*hpaXmΔLP* vectors were constructed [45] (Fig 1a). Screening of T_0_ and T_1_ seed of transgenic tobacco containing the *hpaXm* and *hpaXmΔLP* gene vectors resulted in 35 lines possibly containing *hpaXm* and 43 possibly containing *hpaXmΔLP*. Transformation with the vector pBI121 only and similar screening produced 10 Vec lines.

**Fig 2:**
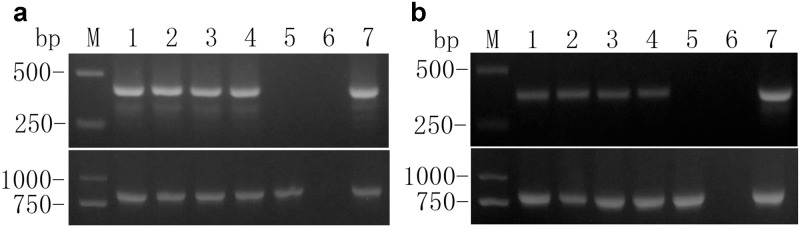
PCR and CaMV35S promoter sequence analyses. Lanes 1–4 are **a)** HpaXm coded on top and CaMV35S promoter coded on bottom from the genome of four randomly selected T_1_ plants of *hpaXm*-transgenic lines and **b)** HpaXmΔLP coded on top and CaMV35S promoter coded on bottom from the genome of four randomly selected T_1_ *hpaXmΔLP*-transgenic plants. Markers (M) are 2000, 1000, 750, and 500 bp. Lane 5 is a negative control of transgenic tobacco genomes with empty vector as template. Lane 6 is a blank control of ddH_2_O as template. Lane 7 is a positive control of plasmids **a)** pBI121::*hpaXmΔLP* **b)** pBI12::*hpaXm* as template.

The *hpaXm* and *hpaXmΔLP* genes were analyzed by PCR, which produced a sequence 100% identical to that deposited in GenBank based on BLAST analyses. The *hpaXm* and *hpaXmΔLP* sequences were detected in transformed callus, regenerated seedlings, and four independent randomly selected *hpaXm*-transgenic plants and four independent randomly selected *hpaXmΔLP*-transgenic plants at various stages, but they were not detected in untransformed and Vec plants (Fig 2). Simultaneously the CaMV*35S* promoter sequence was detected in the randomly selected four independent *hpaXm*-transgenic plants and four *hpaXmΔLP*-transgenic plants, and also in the vector-transformed controls, all of which acquired the predicted sizes around 800 bp (Fig 2). The sequencing results of the PCR-amplified products of the randomly selected transgenic lines, *hpaXm-101* and *hpaXmΔLP-103*, showed that *hpaXm* and *hpaXmΔLP* genes were 100% homologous to the published *hpaXm* sequence (GenBank accession no. **DQ643828**).

**Fig 3:**
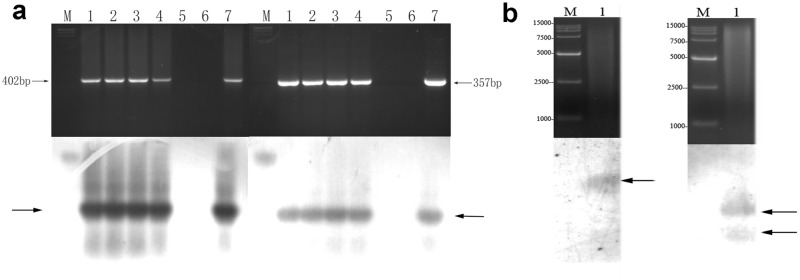
PCR and Southern blot hybridization (bottom) analyses. **a)** Comparison of PCR and PCR-Southern bolt hybridization between the genomes of the full-length *hpaXm*-transgenic plants and the N-terminal leader peptide (1-MNSLNTQIGANSSFL-15) deletion mutant *hpaXmΔLP*-transgenic plants. Lanes 1–4 are four randomly selected T_1_ plants of *hpaXm*-transgenic plants and of the *hpaXmΔLP*-transgenic plants. Lane 5 is the representative Vec transgenic plant T_1_-V35. Lane 6 is of the ddH_2_O as a template. Lane 7 is of the plasmids pBI121::*hpaXm* or pBI121::*hpaXmΔLP* as templates. **b)** Comparison of genomic Southern blot hybridization of representative transgenic plants T_1_-101-3 transformed with *hpaXm* gene (left) and T_1_-103-5 with *hpaXmΔLP* gene (right). Lanes 1 are the genomes digested by enzymes *EcoR*I and *BamH*I coded on top and the genomic Southern blot hybridization coded on bottom. Lane M are the markers. Note: The arrows indicate hybridizing bands.

PCR-Southern hybridization showed that there was at least one copy in the randomly selected four independent *hpaXmΔLP*-transgenic lines and four independent *hpaXm*-transgenic lines. Furthermore, genome Southern blot hybridization confirmed the results, and additionally revealed that one copies of the *hpaXm* trans-gene were present in the *hpaXm-*101 transgenic line, and two copy of the *hpaXmΔLP* trans-gene was present in the *hpaXmΔLP*-103 line. While the Vec control lines and the blank control of ddH_2_O had no visible hybridization bands (Fig 3).

**Fig 4:**
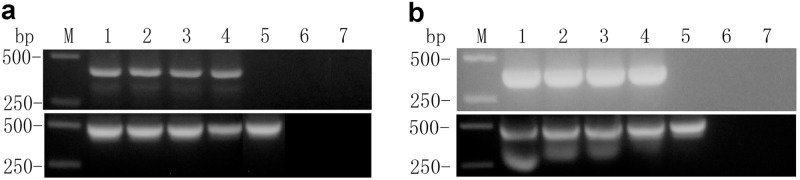
Reverse transcriptional-PCR (RT-PCR) of T_1_ transgenic lines. Results of RT-PCR products detected the expression of the full-length *hpaXm* gene and the N-terminal leader peptide (1-MNSLNTQIGANSSFL-15) deletion mutant *hpaXmΔLP* gene in transgenic lines. Expression of *EFla* gene, a highly conserved and constitutively expressed gene in eukaryotes, used as a quantitative standard. Lane 1–4 are a) of four randomly selected *hpaXm*-transgenic plants and b) of four randomly selected *hpaXmΔLP*-transgenic plants. Lane 5 is the representative Vec transgenic plant T_1_-V35. Lane 6 are a) of the representative *hpaXm*-transgenic plants T_1_-101-3 and b) of the representative *hpaXmΔLP*-transgenic plants T_1_-103-5 without reverse transcriptase. Lane 7 are of ddH_2_O as template. Lane M are the markers.

To determine whether the *hpaXm* and *hpaXmΔLP* inserts were transcribed, RT-PCR was applied to detect accumulation of hpaXm mRNA in the four independent randomly selected *hpaXm*-transgenic lines and hpaXmΔLP mRNA in the four independent randomly selected *hpaXm△LP*-transgenic lines. The *EF1α* gene was used as a standard (Fig 4) because it is highly conserved and constitutively expressed in eukaryotes [46–47]. The sequences of RT-PCR products showed an identity of 100% for *hpaXm* to that deposited in GenBank (GenBank accession no. **DQ643828**) and 99% for *EF1α* to published sequences (GenBank accession no. **DQ174258**, 496 bp). Replicates of RT-PCR performance indicated that *hpaXm* and *hpaXmΔLP* were generated stably from T_0_ to T_1_ generation and expressed normally at the transcriptional level respectively (Fig 4).

Based on height and weight of the entire plant, weight and size of leaves, and flowering time, these botanical traits of the hpaXm-expressing tobaccos, the hpaXmΔLP-expressing tobaccos and the Vec plants evidently resembled the untransformed plants (Fig 1b; and data not shown).

### Soluble proteins produced by transgenic tobaccos lines expressing hpaXm and hpaXmΔLP both elicited HR on tobacco leaves

**Fig 5:**
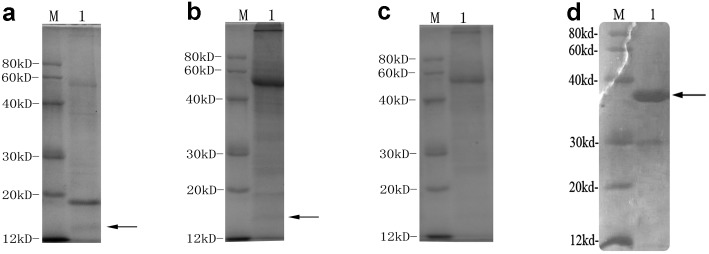
Sodium dodecyl sulfate-polyacrylamide gel electrophoresis (PAGE) patterns of the above preparations. **a)** Soluble protein preparations (1μL; 342ng) including hpaXmΔLP from *hpaXmΔLP*-expressing transgenic plant T_1_-103-5. **b)** Soluble protein preparations (1μL; 368ng) including hpaXm from *hpaXm*-expressing transgenic plant T_1_-101-3. **c)** Soluble protein preparations (1μL; 349.5ng) from Vec-expressing transgenic plant T_1_-V35. **d)** Purified hpaXm (1μL; 264ng) associated with a GST mark.

Note: Arrows indicated the foreign gene expressed proteins of transgenic tobacco. Based on the Bicinchoninic Acid (BCA) Assay method for protein quantitation [48], the protein concentration standard curve was derived through by absorbance of different concentrations of bovine serum albumin. And the protein concentrations were calculated according to the standard curve line Y = 74.9X-11.7. Y: Protein concentration (μg/mL); X: Absorbance values (OD_562_)

To determine the soluble proteins respectively produced by transgenic tobacco expressing the full-length and the signal peptide-like segment-deleted mutant of *hpaXm* genes, we compared sodium dodecyl sulfate–polyacrylamide gel electrophoresis (SDS-PAGE) gel patterns and bioactivity of soluble proteins from leaf tissues of untransformed plants, Vec, and transgenic lines. The putative HpaXm protein contained 133 amino acids, and the molecular mass was estimated to be 13.3 kDa [20]. The putative GST–HpaXm protein was about 35 kDa [20]. The results suggested that a purified protein from the transgenic line was abundant among total proteins and had the same size as hpaXm from *E*. *coli* (Fig 5). Similar results were obtained for *hpaXmΔLP* transgenic lines (Fig 5).

**Fig 6:**
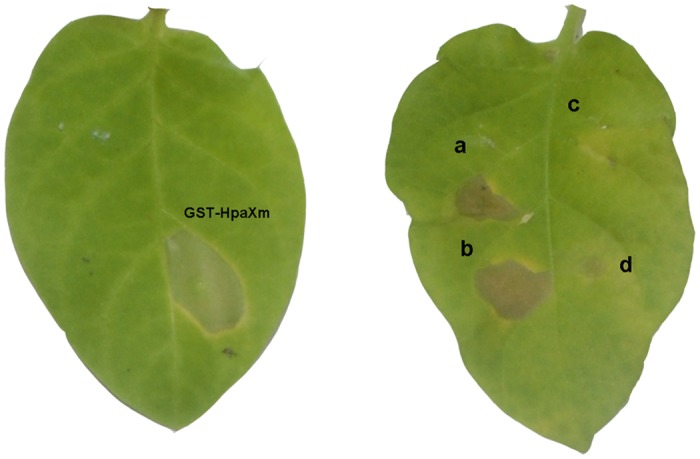
Soluble proteins of the full-length and the signal peptide like segment deletion mutant of hpaXm both could stimulate HR. Bioactivity assays of proteins from leaf tissues, compared with the preparations of GST-hpaXm (0.5μL; 0.13 μg) from *E*. *coli* as control. T_0_ and T_1_ plants of *hpaXm*-expressing lines T_0_-1, T_1_-101-3, and T_1_-101-27, and *hpaXmΔLP*-expressing lines T_0_-7, T_1_-103-5, and T_1_-103-29 were boiled and tested. Data from lines **a)** T_1_-101-3 (1 μL; 0.37μg of boiled soluble-protein preparations) and **b)** T_1_-103-5 (1 μL; 0.34μg of boiled soluble-protein preparations) are shown here. **c)** The Vec line in the T_1_ generation T_1_-V35 (1 μL; 0.35μg of boiled soluble-protein preparations) and **d)** PBS buffer (1 μL) were tested as controls.

We found that only proteins from transgenic plants were active, and extents of the HR they induced in tobacco leaves were comparable with that induced by hpaXm prepared regularly from the recombinant *E*. *coli* strain (Fig 6), even though the proteins were boiled for 10 min. Inversely, proteins isolated from untransformed or Vec plants did not cause the HR (Fig 6). These results demonstrated that either hpaXm or hpaXmΔLP was actively present in leaf tissues of transgenic plants.

### Defensive responses were induced in transgenic tobacco expressing hpaXm and hpaXmΔLP

**Fig 7:**
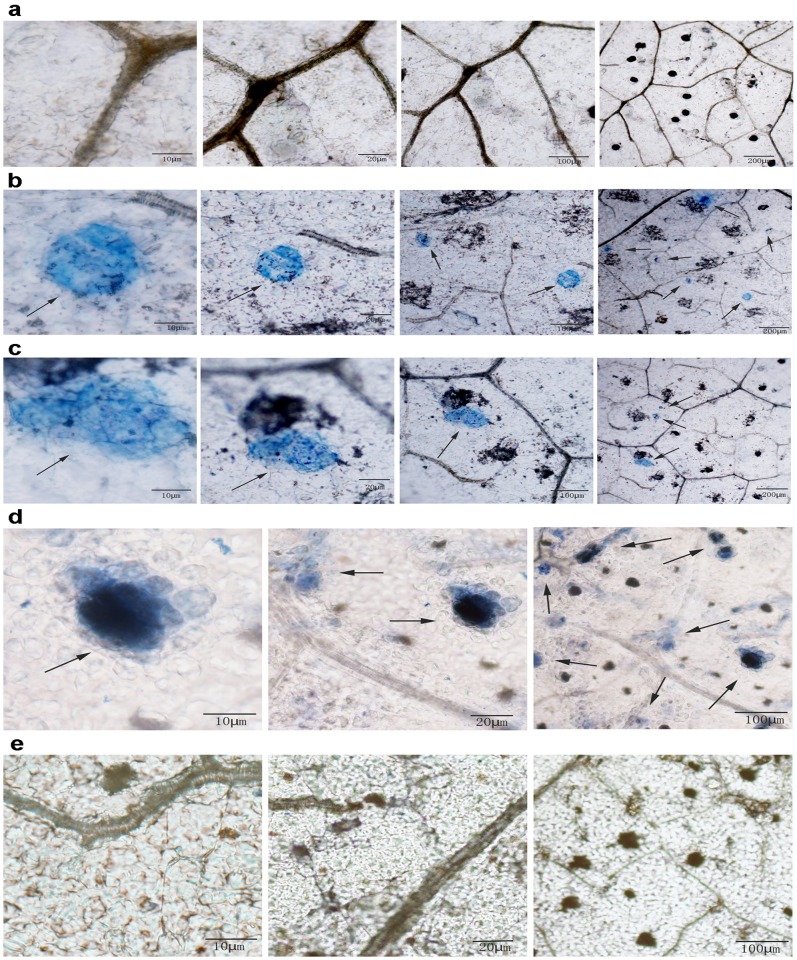
Views of trypan blue-stained leaves of transgenic tobacco plants. Micro-HRs were shown as areas stained blue. Tobacco leaf tissues of the tested transgenic lines, **a)** T_1_-V35 line expressing the vector pBI121, **b)** T_1_-101-3 line expressing the full-length *hpaXm*, and **c)** T_1_-103-5 line expressing the signal peptide-like segment-deletion mutant *hpaXmΔLP*, were stained and observed in the optical microscope under different magnifications. Untransformed tobacco leaf tissues **d)** with exogenous application of the hpaXm proteins as positive control and **e)** with no treatment as negative control were also stained and observed in the optical microscope under different magnifications. Projecting granular lipoglands were not related to any treatment. Note: The arrows indicate the occurrence of micro-HR dead cell clusters.

We observed whole transgenic tobacco plants to determine whether the trans-gene expression of hpaXm induced a defensive response. There was no visible HR or macro-HR on *hpaXm* or *hpaXmΔLP* transgenic tobacco leaf surfaces. However, after trypan blue staining, dark-blue micro-HRs of scattered necrotic cell clusters on leaf tissue were visible under the microscope, indicating that the trans-gene expressed hpaXm and hpaXmΔLP produced micro-HR in transgenic tobacco to varying degrees (Fig 7). The results showed that the trans-gene expression of the full-length and the N-terminal signal peptide-like segment deficient mutant of *hpaXm* produced defensive responses with partial hypersensitive cell death in transgenic tobacco leaves.

**Fig 8:**
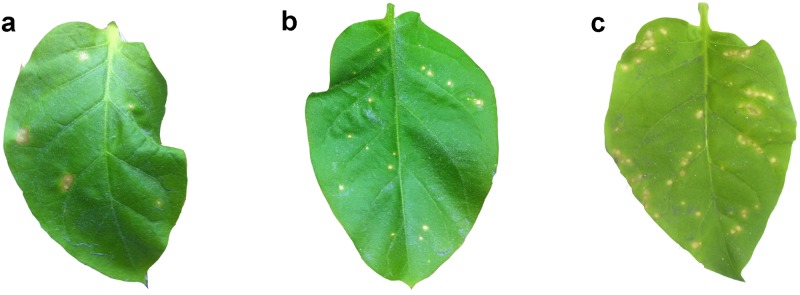
Symptoms of transgenic plantsinoculated with TMV. Photographs of leaves inoculated with 1.8μg TMV, **a)** Leaves of the full-length *hpaXm* transformed line T_1_-101-3, **b)** Leaves of the signal peptide-like segment-deletion mutant *hpaXmΔLP* transformed line T_1_-103-5, and **c)** Leaves of the vector pBI121 transformed line T_1_-V35. Similar results were obtained from five sets of experiments, investigating a total of 67 plants of each genotype.

Furthermore, we evaluated TMV resistance of transgenic lines. Size and numbers of brown spot lesions on leaves caused by TMV were markedly reduced in transgenic plants compared with controls (Fig 8). For example, size and numbers of brown spot lesions developed progressively and expanded by 10 d to basal stems from inoculation sites in untransformed and Vec plants. In contrast, symptoms were restricted around inoculation sites or expanded slightly in transgenic tobacco expressing the full-length and the signal peptide-like segment-deleted mutant of *hpaXm* during the same period. Statistical analysis showed significant differences (*P* ≤ 0.01) between resistant transgenic plants and Vec control plants. Compared with the Vec control plants, the number of lesions on resistant *hpaXm* transgenics was reduced by 60.12% on average, and correspondingly reduced by about 56.67% for resistant *hpaXmΔLP* transgenics (Table 2, Fig 8). The results showed that the trans-gene expression of the full-length and the N-terminal signal peptide-like segment-deleted mutant of *hpaXm* significantly enhanced resistance to TMV in transgenic tobacco.

**Table 2:** Resistance levels of transgenic tobacco against TMV.

Plants	TMV plant lesion number (X±SD)	Significance level (P≤0.01)	Lesion reduction (%)
**The Control**	35.76±2.71	A	
**The transgenic *hpaXm* tobaccos**	12.97±2.37	B	60.12
**The transgenic *hpaXm△LP* tobaccos**	14.16±2.51	B	56.67

### Deletion of signal peptide-like segment prevented the hpaXmΔLP expressed protein moving to plant cell wall

**Fig 9:**
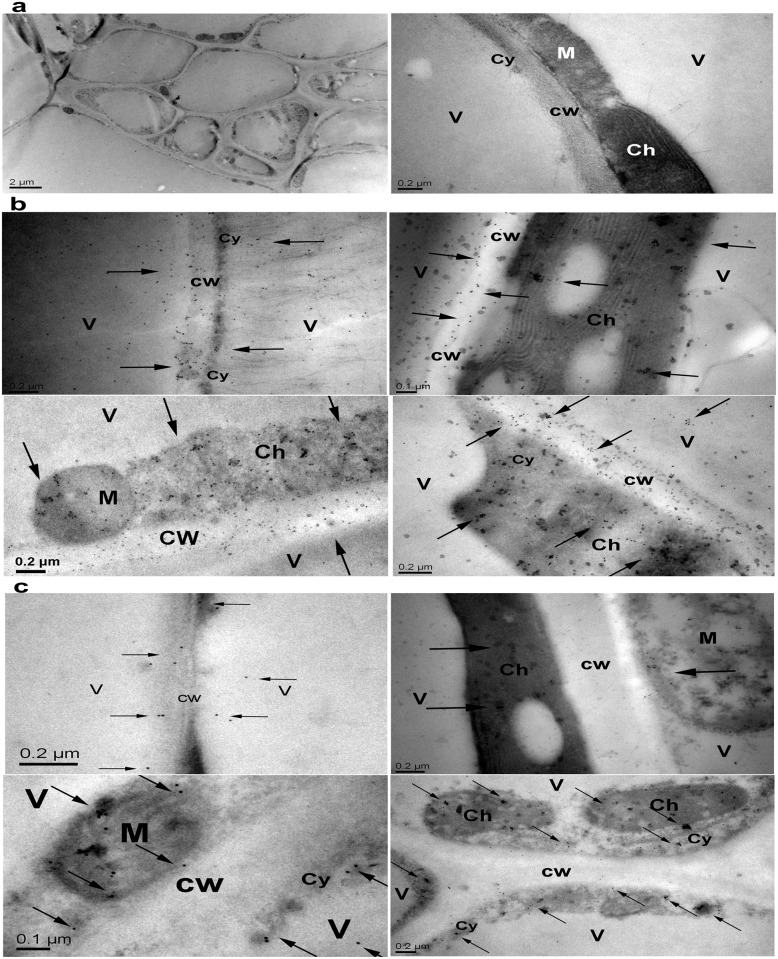
Immuno-gold localization of the full-length and the signal peptide-like segment-deletion mutant of hpaXm in leaves. **a)** Tobacco tissues of vector-transformed T_1_-V35, **b)** tobacco tissues of *hpaXm*-transformed T_1_-101-3, and **c)** tobacco tissues of *hpaXmΔLP-*transformed T_1_-103-5 were pretreatment and observed with a JEM 1200ex transmission electron microscope (Leica, Germany). CW: Cell wall; Cy: Cytoplasm; V: Vacuole; Ch: Chloroplasts; M: Mitochondria. Arrows indicated the colloidal-gold particles (15-nm particles). More than 20 ultrathin sections of each sample were examined. The experiment was repeated twice with similar results.

The immune colloidal-gold tests showed colloidal-gold particles attached to the cytoplasm, the cell membrane, organelles (e.g. chloroplasts, mitochondria, and nucleus), and the cell wall on leaf tissues of every tested transgenic line that expressed the full-length hpaXm. Except for cell walls, colloidal-gold particles were detected in leaf tissues of every tested *hpaXmΔLP* transgenic line as for the full-length lines. For example, colloidal-gold particles were evenly distributed on the plasma membrane of transgenic leaf tissues, and some were clustered in twos and threes. Colloidal-gold particles were densely distributed in the sheet structure of chloroplasts, while patchily distributed in mitochondria at lower density. In particular, colloidal-gold particles were evenly attached to cell walls in *hpaXm* compared with *hpaXmΔLP* transgenic lines. Almost no colloidal-gold particles were observed on leaf tissues in Vec transgenic lines. (Fig 9)

All the tests indicated that hpaXm and its residues were active intracellularly in transgenic lines. Additionally, the exogenously expressed full-length hpaXm was able to move out of the plasma membrane to the cell wall. However, the N-terminal signal peptide-like segment of *hpaXm* deletion resulted in transgene expressed hpaXmΔLP staying inside the plasma membrane.

## Discussion

Unlike other common harpins, HpaXm, is classified in another group in terms of its amino acid composition and some special characteristics [1], such as a putative signal peptide detected in the N-terminal leader peptide [20]. We generated different transgenic tobacco lines expressing the full-length and the N-terminal signal peptide-like segment-deleted mutant of *hpaXm* respectively. Transgenic plants expressing the *hpaXm* and *hpaXmΔLP* genes conferred disease resistance to selected pathogens.

Previously it was reported that several harpins could induce resistance to TMV in tobaccos [49]. In this paper, hpaXm also induced resistance when acting both intercellular and intracellular. Harpin genes are constitutively expressed in plant cells. When sprayed on plants, harpins could confer defense responses without HR cell death against diverse plant pathogens, including fungi, oomycetes, bacteria, and viruses [1]. For example, foliar application of the full-length Hpa1, and particularly the Hpa1_10–42_ fragment, induced strong resistance in rice to *X*. *oryzae* pv. *oryzae* and *Magnaporthe grisea* in greenhouse and field conditions [15,17]. Similarly, transgenic rice expressing *hpa1* showed resistance to *M*. *grisea* [50–51]. Results in the present study supported the view that hpaXm, similar to other common harpins, enhanced the resistance of transgenic tobacco to TMV, even when the N-terminal signal peptide-like segment was deleted. Our data demonstrated that the N-terminal leader peptide (1-MNSLNTQIGANSSFL-15) of hpaXm was not the critical effecter for the resistance. This was similar to the purified hpaXm proteins and the signal peptide-like segment-deletion mutant hpaXmΔLP proteins sprayed on tobacco leaf surfaces [18]. These results showed similar behavior to the harpin Ea, which imparted resistance to *P*. *infestans* [52] and *P*. *nicotianae* [29], regardless of whether *hrpN* was fused with a signal peptide-encoding sequence. All harpins reported so far, except XopA of *X*. *campestris* pv. *vesicatoria* [53] and truncated HrpZ1 of *P*. *syringae* pv. *tabaci* [54], can induce HR in tobacco following infiltration of leaf panels [1]. In this paper, the trans-gene expressed hpaXm and hpaXmΔLP in transgenic tobacco were as active as the recombinant hpaXm and hpaXmΔLP proteins v, being able to elicit HR on tobacco leaves even after they were boiled [20]. Additionally, there was no large area of visible necrotic spots induced in transgenic plants, possibly due to their lower expression level of the foreign genes. However, after staining with trypan blue, micro-HR was observed in transgenic tobacco leaves. The results were similar to the phenotype of transgenic cottons expressing the full-length and partial fragments of harpin_Xoo_ [55]. Firstly, this demonstrated that endogenous expression of hpaXm induced defensive responses in plants with cell death, and the N-terminal leader peptide (1-MNSLNTQIGANSSFL-15) was not sufficient for HR elicitation. Secondly, it demonstrated the heat stability of hpaXm.

There are some controversies concerning the association and interaction sites of harpins. Some evidence demonstrated that several harpins can bind to lipids and form pores in the plant plasma membrane [1], and other evidence indicated that the association site of harpins with plants is also in the cell wall [20, 28, 56–57]. In our previous works, hpa1_Xoo_ were found to accumulate along the cell walls of the transgenic cotton where it could trigger the generation of H_2_O_2_ as a cell wall endogenous elicitor, but only a few found in cell membranes and chloroplasts and none in the mitochondria [21–22]. HpaXm homolog hpa1 is from *X*. *oryzae* pv. *oryzae* [8, 58] and doesn’t own a putative signal leader like hpaXm has [8, 59]. The results of the associations of hpaXm in transgenic tobaccos in this paper show some differences mainly because hpaXm is identified into the group distinct from the Hpa1 group as the glycine ratio of hpaXm is relatively lower than hpa1 and hpa1 carries one cysteine but hpaXm carries four cysteines [1]. Another reason may be different plants used in the experiments withhpa1Xoo in cottons while hpaXm in tobaccos. In addition, the N-terminal leader peptide of several harpins plays an important role in transferring harpins. Therefore, the N-terminal leader peptide of hpaXm probably guided the hpaXm residues transferring from one site to another. Referring to the association sites of the full-length and the N-terminal leader peptide (1-MNSLNTQIGANSSFL-15) deletion mutant of *hpaXm* in transgenic tobacco tissues, deletion of the N-terminal leader peptide segment prevented the hpaXm-deleted residues being transported outside the plasma membrane. The results demonstrated our inference that the N-terminal leader peptide (1-MNSLNTQIGANSSFL-15) of hpaXm guided the residues to associate to the cell wall. Thus, we predicted that hpaXm without the signal peptide could not be translocated to the surface of the leaves.

Previously, association of harpins with the plant plasma membrane was shown by examining the ability of harpins to form pores in artificial membranes, similar to the translocator proteins of animal-pathogenic bacteria [60]; and, additionally, the ability of harpins to bind to lipids. Data from several groups indicated that several harpins can bind to lipids and form pores in the plant plasma membrane [1]. PopA1 from *R*. *solanacearum* was shown to be integrated into liposomes and also into membranes from *Xenopus laevis* oocytes, resulting in formation of ion-conducting pores [27]. In this study, it is not clear why the N-terminal leader peptide of HpaXm is critical for hpaXm transfer to the cell wall. Additionally, how the N-terminal leader peptide or the signal peptide-like segment of hpaXm influences the association sites, the interaction sites and the translocation of hpaXm in plants remains unclear. Study of the association mechanism of hpaXm within host or non-host plants will help us further understand the interactions between harpins, plant pathogens and plants. Thus, more assays of hpaXm binding with plants or pathogens should be done in the future.

## Conclusion

The N-terminal leader peptide (1-MNSLNTQIGANSSFL-15) was not necessary for trans-gene expressed hpaXm to elicit HR on tobacco leaves, to induce micro-HR in transgenic tobacco and to enhance the resistance of transgenic tobacco to TMV. However, the leader peptide was critical for the expressed hpaXm to be transported outside the plasma membranes of transgenic tobacco leaf tissues. Additionally, the N-terminal leader peptide (1-MNSLNTQIGANSSFL-15) of hpaXm acted as a signal peptide in transgenic tobacco, helping hpaXm to associate to the cell wall.

## Open access

The data sets supporting the results of this article are included within the article and its additional files. The microarray data supporting the results of this article are available in the NCBI’s Gene Expression Omnibus repository, and are accessible through GenBank accession numbers DQ643828 (http://www.ncbi.nlm.nih.gov/nuccore/109648387/), AF485783 (http://www.ncbi.nlm.nih.gov/nuccore/AF485783), and AJ223969 (http://www.ncbi.nlm.nih.gov/nuccore/AJ223969).

## Abbreviations

H*rp*: Hypersensitive response and pathogenicity
HR: Hypersensitive response
N-terminal: Nitroxyl-terminal
TMV: Tobacco Mosaic Virus
GST: Glutathione S-transferase
Sec: Sec translocation
Tat: Twin-arginine translocation
T2SS: Type II secretion system
